# Shaping Ability of Superelastic and Controlled Memory Nickel-Titanium File Systems: An In Vitro Study

**DOI:** 10.1155/2018/6050234

**Published:** 2018-09-10

**Authors:** Raidan A. Ba-Hattab, Dieter Pahncke

**Affiliations:** ^1^Department of Clinical Dental Sciences, College of Dentistry, Princess Nourah Bint Abdulrahman University, Riyadh, Saudi Arabia; ^2^Department of Operative Dentistry and Periodontology, Dental School, University of Rostock, Rostock, Germany

## Abstract

Improvements in the thermomechanical processing procedures of NiTi wires have led to the development of new NiTi instruments that compose mainly of martensite crystals, making the wire stable at clinical condition. This study aimed at comparing the shaping ability of two rotary nickel-titanium systems manufactured from different NiTi wires. Twenty simulated root canals each with a curvature of 35° in resin blocks were divided into two groups of 10 canals each. Canals in the first group were prepared with superelastic F360 instruments (Gebr. Brasseler, Germany) while canals in the second group were prepared using controlled memory HyFlex®CM™ instruments (Coltène Whaledent, Switzerland). Images were taken before canal preparation and after the use of each instrument. The assessment of the canal shapes was accomplished with a computer image analysis program. Data were statistically analyzed using SPSS program. Within the limitation of this in vitro study, HyFlex®CM™ instruments remained better centered in the apical third of the canals. In most canal segments, no significant differences were observed between either system in the amount of material removed. Both systems were comparable to each other in regards to their ability to enlarge root canal in the same way without procedural errors.

## 1. Introduction

During the last decade, many dental companies have been directed for manufacturing of different NiTi rotary instruments with different designs including noncutting tips, radial lands, different cross sections, different helical angle, and varying tapers with the aim to improve their performance and to simplify the preparation procedure [1]. Recently, thermal treatment of NiTi alloy is frequently used [2, 3] to further increase flexibility and fatigue resistance of rotary NiTi instruments rather than changes in instrument geometry [4].

HyFlex®CM™ NiTi files (Coltène Whaledent, Switzerland) are made from an innovative thermomechanical process of NiTi alloy with the property of “Controlled Memory” rather than “Superelastic property” of other conventional NiTi files [4]. These instruments are in the martensite condition at body temperature [5]. Instruments with sizes 20/0.02, 20/0.06, 30/0.04, and 40/0.04 have triangular cross section with three blades and three flutes, other instruments with sizes 20/0.04 and 25/0.04 have quadrangular cross section with four blades and four flutes [6].

The recent F360 system (Komet Dental, Lemgo, Germany) is a 2-file system. The instruments have a 4% taper and are available in sizes 25, 35, 45, and 55. They have a modified double S-shaped cross section and are made of conventional, superelastic NiTi alloy [7].

The null hypothesis was that there would be no difference between superelastic, conventional NiTi instruments (F360) and controlled memory NiTi instruments (HyFlex®CM™) regarding their shaping ability in simulated root canals.

## 2. Materials and Methods

### 2.1. Resin Blocks and Experiment Design

A total of twenty transparent canals made of clear polyester resin (Endo Training Block 02 taper, REFA 0177; Dentsply Maillefer, CH-1338 Ballaigues, Switzerland) were used in this study. All canals had an apical foramen of 0.15 mm, a taper of 0.02, and an angle of curvature of 35°. Canal length was 17 mm with a straight section of 12 mm and a curved section of 5 mm. The samples were randomly divided into two experimental groups (*n*=10). Using a diamond bur, a small hole was drilled on one side of the preinstrumented block to ensure superimposition accuracy of pre- and postinstrumentation canal pictures during subsequent image analysis. Then, red solution (Caries Marker, coloured caries indicator, VOCO, Cuxhaven, Germany) was injected into the canals to recognize them easily from the postinstrumented canal.

To secure a stable position of the resin blocks, a metal holder was made in which the resin blocks could be placed and repositioned in exactly the same position. A digital camera EOS 400 Digital (Canon Inc., Tokyo, Japan) with a macro-objective “Tamron SP AF 60 mm F/2 Dill Macro 1 : 1” (Tamron Co., Ltd., Saitama, Japan) was used in a fixed position to capture pictures before and after canal instrumentation, and the pictures are saved directly as JPEG format files in a computer. A black background was placed behind the blocks, and the simulated canals were prepared with any of the two systems: F360 and HyFlex®CM™.

### 2.2. Preparation of the Simulated Canals

The instruments were set into CanalPro CL cordless motor handpiece (Coltène Whaledent, Switzerland) with contra-angle head of 16 : 1. Torque limits and the rotational speeds of each file which recommended by the manufacturers were entered and stored manually by the operator. The instrumentation of all blocks was carried out by one experienced operator.

#### 2.2.1. Group 1: Superelastic Group

Root canals were prepared using F360 instruments (Gebr. Brasseler, Germany). File sizes 25/0.04 and 35/0.04 were used in a single-length technique at a constant rotational speed of 300 rpm and a torque-control level of 1.8 N·cm as recommended by the manufacturer. The instruments were placed in the canals sequentially with a gentle picking motion.

#### 2.2.2. Group 2: Controlled Memory Group

Root canals were prepared using HyFlex®CM™ System (Coltène Whaledent, Switzerland). The instruments were used in a single-length technique at a constant rotational speed of 500 rpm and a torque-control level of 2.5 N·cm as the suggested settings by the manufacturer. To standardize the apical preparation, two files of HyFlex®CM™ (25/0.04 and 35/0.04) were used to prepare the canals instead of conventional full sequence. The files were also placed sequentially in the canals with a gentle picking motion.

Each instrument was coated with FileCare (EDTA, VDW, München, Germany) to lubricate the canals during instrumentation, and a total of 5 ml water was used repeatedly after the use of each instrument. Each instrument was used to shape one canal only. Once the instrument had reached the full working length and rotated freely, it was removed.

### 2.3. Assessment of Canal Preparation

**Figure 1:**
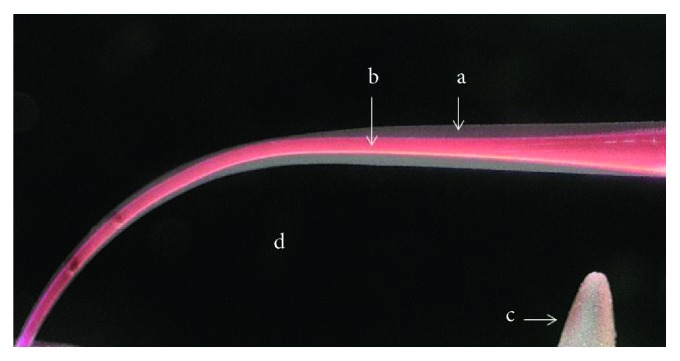
A composite image of the simulated canal from the HyFlex CM group (a) after instrumentation (white area) and (b) before preparation (red area) with (c) black background and (d) a drilled hole to secure superimposition of the canals.

**Figure 2:**
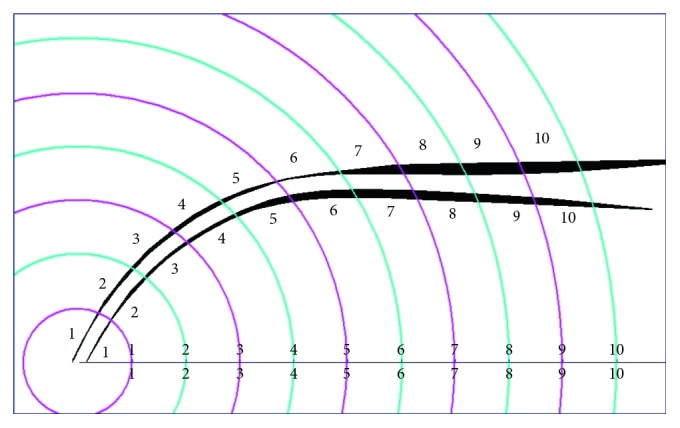
20 segments (10 segments in the inner wall and 10 segments in the outer wall) are created by the ten concentric circles (F360 group).

Image analysis software (GSA Image Analyser Software development and Analytics Bansemer and Scheel GbR, Germany) was used for the assessment of canal curvature modification. The pictures of the simulated canals before and after instrumentation were superimposed using the software, producing a composite image for each canal (Figure 1). The area between canal walls before and after instrumentation was determined both for the inner and outer canal curvature using the same image software. The composite image was sectioned by ten concentric circles spaced 1 mm apart. The centers of the circles were targeted over the tip of the preinstrumented canal, i. e., the first circle radius was 1 mm from the canal tip, and the last circle radius was 10 mm from the canal tip, resulting in 10 measuring segments on the outer and inner sides of the canal, for a total of 20 measuring segments (Figure 2). These segments (material removed) were measured as a surface area (mm^2^) automatically using the GSA Image Analyser program.

Moreover, procedural errors occurred during instrumentation described by Thompson and Dummer [8] were also assessed based on the composite images.

### 2.4. Data Analysis

All data were recorded and statistically analyzed using SPSS (version 19.0, IBM Corporation, USA). The significance level was set at *P* ≤ 0.05. The Wilcoxon test was used to compare the area removed from the inner and outer canal walls of one group. The Kruskal–Wallis test was used to compare canal transportation between the groups.

## 3. Results

The mean values and standard deviations of the area removed from the inner and outer curvature of the canals are detailed in Table 1. The two systems removed significantly (*p* ≤ 0.05) more material on the outer wall than the inner wall in the coronal part of the canal (segments 8–10). In the middle part of the canal (segments 5–7), more material was removed on the inner wall than the outer wall; the difference was statistically significant (*p* < 0.05) except in segments 5 and 7 of F360 and HyFlex®CM™ groups, respectively. Apically (segments 1–4), the difference between the material removed from the inner and outer canal walls was not statistically significant in segment 2 of F360 group and in segments 1 and 2 of HyFlex®CM™ group.

There was no statistically significant difference between the F360 and HyFlex®CM™ groups in the mean material removed from the inner and outer wall of the canals (Table 2), except in two segments (8 and 9) on the inner wall and only one segment (8) on the outer wall (*p* ≤ 0.05).

Regarding procedural errors, no loss of working length or canal aberration was recorded during canal instrumentation in any of the groups.

## 4. Discussion

The purpose of this study was to compare the shaping ability of two NiTi endodontic instruments manufactured from different NiTi wires: superelastic NiTi instruments (F360) and controlled memory NiTi instruments (HyFlex®CM™) in simulated root canals in resin blocks. HyFlex®CM™ instruments are manufactured from a special NiTi alloy that has been claimed to have a lower percent in weight of nickel **52%** [9] and subjected to thermomechanical processing that creates a mixture of martensite and austenite structures [5].

In this study, simulated canals in resin blocks had been used as an experimental model for assessment of shaping ability of the instruments. These simulated canals are an alternative to the real human extracted teeth and have been used in several studies to test the shaping ability of files [10, 11]. Although the major advantage of using extracted human teeth is the reproduction of the clinical situation, the variations in three-dimensional root canal morphology makes standardization difficult [12]. Resin blocks provide standardized experimental conditions but may not fully represent the clinical settings as they have several limitations related to their mechanical properties which differ from human dentin and heat generation during instrumentation which might lead to instrument separation [13].

Standardization of the apical end preparation is essential to compare the shaping ability of different root canal instruments [14]. In this investigation, the apical end of the canals was prepared using instruments.

According to the results of this study, HyFlex®CM™ and F360 systems did not remain perfectly within the center of the root canal and showed canal straightening toward the inner wall in the middle third and toward the outer wall apically and coronally. Centering ability of the instruments is influenced by several parameters such as instrument design and alloy from which the instrument is manufactured. Instruments with small cross-sectional designs proved better centering ability [15] as the minimal amount of the residual core improves instrument flexibility [16]. Although HyFlex®CM™ instruments have a larger cross-sectional design (quadrangular and triangular cross section for size 25/0.04 and 35/0.04, respectively) in comparison with the small, modified S-shaped cross section of F360 instruments, the instruments remained better centered apically than F360, where nearly the same amount of resin material was removed from the first and second segments of the canal. This might be explained by the good flexibility of the unique, controlled memory (CM) wire of HyFlex®CM™ instruments. Furthermore, the elongation of their spirals during canal preparation allows better removal of debris from the canal [17]. F360 instruments are manufactured from conventional, superelastic NiTi alloy, and this means that the instrument straighten itself while preparing curved canal and attempts to regain its original shape which result in uneven stress on canal walls and consequently uneven material removal from the canal [18].

Recently, Gu et al. [19] stated that the alloy type of the instruments influenced canal transportation more than their cross-sectional designs (*p* < 0.05), and the CM-wire based instruments created the most favorable preparations amongst the thermally treated NiTi instruments in resin canals.

The results of the present study are in agreement with those reported by Bürklein et al. [20], who used the same way as in this study to compare five systems: WaveOne (Dentsply Maillefer, Ballaigues, Switzerland), Reciproc (VDW), OneShape (Micro-Mega), HyFlex®CM™, and F360. They found less transportation in canals prepared with F360, OneShape, and HyFlex®CM™ when compared with instruments of reciprocating motion. Similar conclusions were reached by Rashid and Saleh [21], who compared WaveOne, Reciproc, OneShape, and F360. They concluded that all systems maintained root canal curvature well and were safe to use, and canals prepared with the F360 and OneShape systems were better centered compared with the Reciproc and WaveOne systems.

James et al. [22] stated that HyFlex®CM™ files produce less canal transportation when compared with other thermally treated NiTi Files. A study by Łęski and Radwański [23] showed that HyFlex®CM™ files are more flexible than ProTaper Next® (Dentsply Maillefer, Ballaigues, Switzerland). Another study showed that Hyflex CM instruments resulted in significantly less canal straightening as compared to the use of ProTaper Universal [24].

In summary, the current study showed that both HyFlex®CM™ and F360 instruments prepared canals without significant shaping errors and there was no significant difference between them. Therefore, the null hypothesis was accepted.

## 5. Conclusions

Within the experimental limitation and the results of the present study, it could be concluded that both systems were comparable to each other in regard to their ability to enlarge root canal in the same way without procedural errors.

## Figures and Tables

**Table 1:** Area removed^1^ (mm^2^) for each instrument.

Segments	1	2	3	4	5	6	7	8	9	10
*F360*										
Outer wall	0.03 ± 0.01	0.04 ± 0.02	0.06 ± 0.02	0.07 ± 0.02	0.07 ± 0.02	0.05 ± 0.02	0.08 ± 0.03	0.13 ± 0.02	0.16 ± 0.03	0.16 ± 03
Inner wall	0.02 ± 0.01	0.03 ± 0.01	0.03 ± 0.01	0.04 ± 0.02	0.09 ± 0.03	0.13 ± 0.03	0.12 ± 0.02	0.10 ± 0.01	0.10 ± 0.01	0.09 ± 02
*Pvalues*	**0.047** ^*∗*^	0.396	**0.007** ^**∗**^	**0.011** ^**∗**^	0.128	**0.005** ^**∗**^	**0.005** ^**∗**^	**0.005** ^**∗**^	**0.005** ^**∗**^	**0.005** ^**∗**^

*HyFlex®CM™*										
Outer wall	0.03 ± 0.01	0.03 ± 0.02	0.06 ± 0.02	0.07 ± 0.02	0.05 ± 0.02	0.04 ± 0.01	0.08 ± 0.01	0.11 ± 0.02	0.14 ± 0.02	0.15 ± 0.02
Inner wall	0.03 ± 0.01	0.04 ± 0.02	0.03 ± 0.01	0.05 ± 0.02	0.10 ± 0.02	0.13± 0.02	0.10 ± 0.02	0.08 ± 0.01	0.08 ± 0.02	0.07 ± 0.02
*Pvalues*	0.096	0.726	**0.008** ^**∗**^	**0.015** ^**∗**^	**0.009** ^**∗**^	**0.005** ^**∗**^	0.076	**0.009** ^**∗**^	**0.007** ^**∗**^	**0.005** ^**∗**^

^1^Mean ± standard deviation; ^*∗*^values are statistically significant.

**Table 2:** Comparison between the instruments of the area removed^2^ (mm^2^) from canal walls.

Segments	1	2	3	4	5	6	7	8	9	10
*Outer wall*										
F360	0.03 ± 0.01	0.04 ± 0.02	0.06 ± 0.02	0.07 ± 0.01	0.07 ± 0.02	0.05 ± 0.02	0.08 ± 0.03	0.13 ± 0.02	0.16 ± 0.03	0.16 ± 0.03
HyFlex®CM™	0.03 ± 0.01	0.03 ± 0.02	0.06 ± 0.02	0.07 ± 0.02	0.05 ± 0.02	0.04 ± 0.01	0.08 ± 0.01	0.11 ± 0.02	0.14 ± 0.02	0.15 ± 0.02
*P values*	0.812	0.439	0.592	0.585	0.301	0.133	0.319	**0.017** ^**∗**^	0.055	0.135

*Inner wall*										
F360	0.02 ± 0.01	0.03 ± 0.01	0.03 ± 0.01	0.04 ± 0.02	0.09 ± 0.03	0.13 ± 0.03	0.12 ± 0.02	0.10 ± 0.01	0.10 ± 0.01	0.09 ± 0.02
HyFlex®CM™	0.03 ± 0.01	0.04 ± 0.02	0.03 ± 0.01	0.05 ± 0.02	0.10 ± 0.02	0.13 ± 0.02	0.10 ± .002	0.08 ± 0.01	0.08 ± 0.02	0.07 ± 0.02
*Pvalues*	0.218	0.908	0.540	0.966	0.254	0.909	0.086	**0.021** ^**∗**^	**0.019** ^**∗**^	0.066

^2^Mean ± standard deviation; ^*∗*^values are statistically significant.

## Data Availability

The data referring to the area removed from each sample which are used to support the findings of this study are available from the corresponding author upon reasonable request.
